# Systematic Review of Integration and Radicalization Prevention Programs for Migrants in the US, Canada, and Europe

**DOI:** 10.3389/fpsyt.2021.606147

**Published:** 2021-07-29

**Authors:** Natalia Del Pino-Brunet, Isabel Hombrados-Mendieta, Luis Gómez-Jacinto, Alba García-Cid, Mario Millán-Franco

**Affiliations:** ^1^Department of Social Psychology, Social Work, Social Anthropology and East Asian Studies, University of Málaga, Málaga, Spain; ^2^Faculty of Social and Labour Studies, University of Málaga, Málaga, Spain; ^3^Faculty of Psychology, University of Málaga, Málaga, Spain

**Keywords:** migrants, integration programs, prevention of radicalization, intervention, assessment, systematic review

## Abstract

**Background:** International migration processes are some of the most important events of our time. Migrating implies a broad range of factors that affect integration, and which may be linked to radicalization. Host countries use different methods for the integration of migrants. The aim of this systematic review is to analyze intervention programs that aim at promoting social integration and preventing the radicalization of migrants, with the objective of studying actions carried out in the US, Canada, and Europe to this effect.

**Method:** Worldwide known bibliographic databases (PsyCINFO, Sociological Abstracts, Psycarticles, Psychology Database, Medline, SCOPUS, and Google Scholar) were used to search studies published before January 2019 and which analyzed integration or radicalization prevention programs with migrants.

**Results:** A total of 601 studies were screened, from which 18 met the inclusion criteria. The analysis of programs addressed to migrant women points to a decrease in loneliness and depression and an increase of migrants' perception of social support and self-esteem. Programs carried out in schools and families improve children's knowledge of their culture and that of others, increase their feelings of inclusion, and reduce their aggressiveness, stress, and anxiety. Language programs promote communication and employment search, as well as improve migrants' quality of life. One of the most effective activities included in these programs is to connect recently arrived migrants with local population and/or long-term residents. The latter act as mentors and teachers, helping recently arrived migrants understand resources and to be more integrated in the new country, as well as reducing discrimination from the local population.

**Conclusions:** The review concluded the importance of intervention programs for integration, migrants' quality of life, prevention of extreme behaviors, and intercultural cohabitation. Future programs must be more detailed regarding participants' information and carry out more comprehensive assessments.

## Introduction

The phenomenon of migration is universal. According to Blackwell (1), humans have always migrated to different territories in search of security, resources, and ideological freedom, and to escape wars and political persecution, among others, with the objective of improving and even saving their lives. The movement of people to other areas, both voluntarily and forced, through country borders is an expression of globalization that affects national, economic, and political stability as well as cultural identities (2, 3). The number of forcedly displaced people worldwide increased more than 2.9 million in 2017, and by the end of the same year, more than 70 million people had forcedly fled their homes (4). According to the IOM (International Organization for Migration) (5), there was an increase in migration and displacements in 2018 caused by conflicts, persecutions, degrading situations, and climate change, and a strong lack of opportunities and security. As a result, international migration processes are one of the most important phenomena of our time, with a high interest in such phenomenon and the people implied in it. Arango (6) mentions that there are still numerous obstacles in the understanding of this situation due to the diversity of processes, motivations, socio-economic and cultural contexts, etc., thus hindering the creation of theories in this field. In this sense, Mora (7) notes that the new current spaces and forms of migration make the study of their dimensions difficult, requiring a high-level interdisciplinary methodology. Additionally, restrictive migration policies cause higher levels of illegal migration and, therefore, the exact numbers of this collective is harder to calculate (8). This is why, in the year 2020, the International Organization for Migration (OIM) highlighted the need to convey research on migration as a key function of the Organization. This means that Member States using diverse data and research and analysis methods will receive support from the Organization in the creation of migration policies.

The conceptualization of social integration processes for migrants is a complex matter and there is a lack of consensus. The variety of approaches from which integration has been analyzed is explained by the interest shown by several disciplines, such as Sociology, Anthropology, Social Psychology, and Demography, among others (9). Furthermore, according to each country, there are differences when addressing this phenomenon. The European Union is developing a common approach in terms of migration in order to tackle the challenges posed by this type of international mobility (10). Conversely, the increasing arrival of migrants in the US, according to Pérez (11), forced social service and mental health systems to create new services, sensitivities, and interventions for large groups of people with acknowledged necessities.

Migration implies a wide variety of factors that affect the integration of individuals from different origins and situations. Migration does not generally take place under favorable circumstances; on the contrary, most migrants face huge challenges upon their arrival in host countries: securing a place to live, overcoming language barriers, finding employment, and adapting to new systems and expectations. They also face physical, legal, structural, and social barriers that affect their arrival and subsequent integration (12). Separation from the family, their homes, and countries, as well as social, cultural, and economic problems are also linked to migration processes (13). Bhattacharya (14) adds other factors such as facing loneliness (lack of family environment), frustration (inability to success), and lack of control over employment conditions.

Failed integration of migrants can lead to serious consequences for host countries. According to Holguín (15), a balance between family and school education, access to employment, access to decent housing, and participation in the host society are signs of cohabitation and integration. Conversely, when this is not achieved, coexistence and confrontation take place. Gómez (16) notes that the lack of resources or social status increases risky behaviors in migrants in order to obtain what it is wished for. Under insecure environments, individuals who perceive exclusion and feel disconnected from society may “turn their backs on society” and look for an alternative group with extreme ideas (17). Studies point out that personal traumas, shame, humiliation, and abuse perceived is linked to higher racism (18). Therefore, the integration of migrants must be a top priority for host countries.

The increasing number of migrants in Europe, US, and Canada makes it necessary to develop intervention programs aimed at integrating migrants and stopping radicalization processes. Based on this statement, we ask ourselves the following:

*Which intervention programs are being carried out to favor integration and prevent the radicalization of migrants?*

### The Present Study

The objective of the present study is to update and analyze intervention programs aimed at migrants' social integration in order to know the actions that are currently being carried out in this field. More specifically, we want to analyze the radicalization prevention programs in place. For this purpose, a systematic review of empirical studies that contributed to the development of programs for integration and/or prevention of radicalization of migrants was suggested. The review was performed following the PRISMA recommendations for information search and report processes (19).

## Materials and Methods

### Design

Systematic review of programs published in scientific papers put in place to promote migrants' integration and prevent radicalization.

### Initial Search

In order to achieve the objective of the research, a systematic search of scientific papers was carried out in two stages. Seven databases were consulted in total. Initially, searches in worldwide known databases were carried out (PsyCINFO, Sociological Abstracts, Psycarticles, Psychology Database, Medline, SCOPUS, and Google Scholar). ProQuest metasearch engine was used in the first stage to group the first five databases. The first search was conducted on December 11, 2018, and the second one was conducted on January 11, 2019, for confirmatory and updating purposes. According to the information extracted, the search was broadened to the remaining two databases, carried out between February 4 and 8, 2019. Terms used for each database and the number of papers that met the initial filtering are shown in Table 1. PRISMA recommendations were followed, consisting of a list of 27 indicators considered key for the process of registering and reporting systemic reviews and meta-analyses (19).

**Table 1:** Filtering process and inclusion criteria.

**Terms used for the search based on databases and initial results**		
**Databases**	**Search terms**	**Initial results**
ProQuest (PsycInfo, Sociological Abstracs, PsycArticles, Psychology Database, Medline).	(intervention) AND (integration) AND (immigration) AND (program) (Evaluation program) AND (immigrants) AND (intervention) (Immigration program) AND (evaluation) AND (integration) (Intervention program) AND (immigrants) AND (evaluation) (“muslims” AND (“prevention programs”)) (“muslims” AND (“intervention”) AND (evaluation programs)) (“muslims” AND (“teenagers”) AND (programs)) (“muslims” AND (“teenagers”) AND (integration programs))	397
SCOPUS	(programs) AND (immigrants) AND (evaluation) AND (integration) (“muslims” AND (integration) AND (program))	68
Google Scholar	Integration program with immigrants Programs radicalization prevention Muslims	136
Total		601

**Figure 1:**
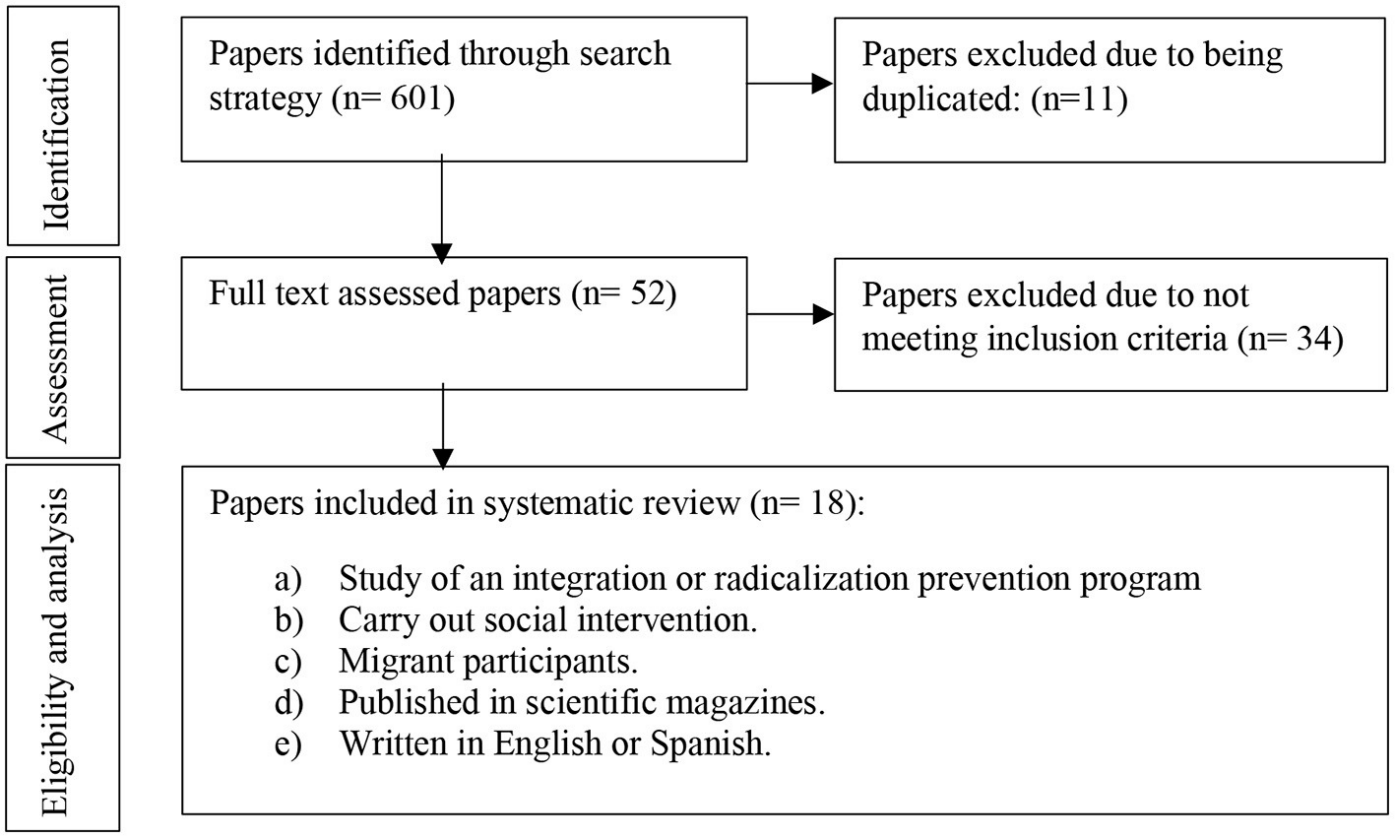
Flow diagram based on PRISMA declaration (19).

The initial screening process consisted in locating title, abstract, and participant information with the aim of verifying whether the following inclusion criteria were met: (a) study of an integration or radicalization prevention program, (b) study that includes social intervention, (c) migrant participants, (d) papers published in magazines, and (e) written in English or Spanish. When in doubt, if a paper met or not some of the criteria, they were included to be screened in the stage of full-text analysis. A total of 601 papers were screened. Of those papers liable to be included, 11 were found duplicated in some databases. Those papers that did not meet inclusion criteria were removed from the remaining 590. Most papers did not meet criterion b, meaning that they did not include social intervention with the collective (Figure 1).

### Full-Text Analysis

Fifty-two papers met the inclusion criteria to be included in the full-text analysis (35 from ProQuest, 13 from SCOPUS, and four from Google Scholar). Thirty-three papers were discarded, most of them due to not including interventions but research through questionnaires to know the factors that affect migrants or about integration policies. One paper was discarded due to being written in French. The remaining 18 papers met the criteria and passed to the review stage. Papers were read by one researcher and codified according to categories described in Table 2.

**Table 2:** Categories to register papers for review.

**Categories**	**Description**
Location	Geographical location.
Duration	Length of the program.
Funding	Funds received by the program.
Participants	Characteristics of the program's recipients (gender, origin, and range of age or average age of participants).
Objetives	Situation that is desired to be obtained as a result of specific actions once program is ended.
Characteristics of the Intervention	Type of techniques used and/or activities put in place to achieve objectives.
Assessment methods	Instruments used for assessment (interviews, questionnaires, follow-up of participants, etc.)
Results	Goals achieved, effectiveness of the program, and participants' satisfaction.

## Results

The following categories were considered for the analysis of results: Location of the program, duration, funding, participants, objectives, characteristics of the intervention, assessment method, and results (see Table 2). Such categories were chosen due to the specific relevance of each of them in our research. They represent the fundamental and essential features of the majority of programs reviewed, thus allowing us to analyze them and determine whether interventions worked appropriately.

The following assessments were concluded after analyzing each category named above.

### Countries That Carry Out Integration Programs for Migrants

Results show that there are six integration programs in the US [see list of references (1, 2, 3, 4, 5, 6)], nine in Europe (7, 8, 9, 10, 11, 12, 13, 14, 15), and three in Canada (16, 17, 18). All programs focus on the integration of migrants.

### Participants

In most programs, participants come from Latin America (1, 2, 3, 5, 6, 10, 11, 18), followed by Arab countries (5, 8, 12, 15, 16, 17, 18) and European countries such as Greece and Russia (7, 12). Some programs do not specify the origin of migrants (4, 9, 13, 14).

A large proportion of programs are addressed to migrant women (2, 5, 6, 10, 11, 12, 16). This is probably because there has been an increase in the number of migrant women, according to the General Assembly of the United Nations (20), who represent almost half of the 244 million migrants and half of the 19.6 million refugees worldwide. Women's vulnerability in migration is called “feminization of migration,” due to the existing inequalities that make causes and consequences of migration to be different for men and women (21). From a gender approach, migrant women face higher discrimination, insecurity, abuse, and violence (22, 23). Furthermore, these women are in host countries that do not really provide opportunities for social mobility and improvement of their quality of life, which prolong these women's social vulnerability situations (24). Generally, jobs found by migrant women hinder their integration—almost one in every six domestic workers worldwide are international migrants, women representing 73.4% of the total domestic workers who are international migrants (25). To such an employment situation, the gender violence experienced by migrant women, particularly Latin women, must be added, as well as the existence of psychological disorders such as low self-esteem, lack of support networks, and difficulties to adapt to the new environment (26). As a result, most of the programs found, which includes interventions with women, aim at empowering them and improving their self-esteem (2, 11, 12, 16), reducing their stress levels and depression (6, 10), and promoting support networks in the community.

Other programs are addressed to recently arrived migrants (4, 8), since the process of integrating in the new country is usually a challenge due to integration barriers (27). There are also programs addressed to unemployed migrants (14). Some studies show that, in general, unemployment rates in migrants are higher than the local and native population in the EU (28).

Finally, programs addressed to children's and adolescents' integration in the school environment must be noted (1, 7, 9, 13, 18). These programs are very relevant because studies show that migrant students who have low academic performance relate to negative self-esteem, stress, insecurity, etc. (29–31).

### Length of Intervention Programs

In the US, most programs are long term. There are two with a duration of 3 years (4, 6), one with a duration of 2 years (3), and another one with a duration of 1 year (5). Conversely, Europe carries out short-term programs (10 weeks, 3 months, 5 months, and 6–8 months). Except for one intervention that has a duration of 6 years (9), the remaining ones have a duration of <1 year. In Canada, the lengthiest program has a duration of 6 months (16).

### Funding of Programs

The programs analyzed focus on issues that, as pointed out by Suárez-Orozco and Carhill (32), are four broad urgent fields of interest for the psychosocial situations of migrants: (a) acculturation stress and “migratory morbidity,” (b) tensions related to family dynamics, (c) challenges for the creation of identities, and (d) educational adaptation and results. The objectives of the programs will be differentiated based on this classification system.

### Acculturative Stress and “Migratory Morbidity”

One of the studies analyzed focuses on acculturative stress and “migratory morbidity” (3). This study focuses on the integration of migrants in the health system in order to ensure healthcare is accessible and linguistically adapted to them. Some authors confirm that migrating can modify health, not because of the experience on its own, but due to the conditions under which it takes place (33). According to Carpentier and de la Sablonnière (34), a better understanding of the specific challenges faced by migrants is useful for health officials' daily practices. It is important to note that petitions for cultural competencies arose from the concern of States about minoritarian ethnic populations. These feelings of concern appeared based on the studies carried out, which revealed that mental health services were not accessible and were not being provided effectively to such populations (35).

### Tensions Related to Family Dynamics

One of the programs analyzed that focuses on the tensions related to family dynamics aims at improving the skills of Muslim parents (15). It is important to highlight that this program suggests tackling parents' emotional and cognitive issues by teaching them positive skills to educate their children. This has a positive impact on children and makes them grow in a calmer and happier environment. It is remarkable that it took 1 year to adapt this intervention in order to make it compatible with Islamic teachings and make discussion subjects in line with Mohammed's actions. In general, it has been proved that improving parents' skills to face their children's education has a positive impact for the family's functioning (36). In this field, another program can also be included, which includes parents in order to improve students' integration with the objective of training them in the education of their children and family communication (1).

### Challenges for the Creation of Identities

In the field of the challenges for the creation of identities, we found programs that aim at integrating migrants based on language skills (8, 14). Linguistic differences prevent many migrants from participating in the host society and integrating in the new community. Studies show that for recently arrived migrants, knowing the language and the culture of the host country helps them overcome the barrier of communication as well as makes it easier for them to find employment (37). Authors such as McMichael and Manderson (38) note that networking sites allow access to information, goods, and services of the host country and help migrants keep their bonds with their places of origin. As migrants search for a long-term place to settle in host countries, their networks are essential to allow their integration and maintain their cultural identity (12). According to Suárez-Orozco and Carhill (32), support networks play a key role in maintaining and improving self-esteem, as well as providing acceptance, approval, and sense of belonging.

### Educational Adaptation and Results

The programs that carry out educational adaptations and results aim at improving migrants' social skills, empathy, acceptance, ability to express feelings, and students' empowerment (7, 18). Studies show that adolescents with high levels of global self-concept show behaviors of self-control and leadership and low levels of shyness, isolation and anxiety, as well as good levels of social adjustment and improved chances of making friends and classmates (39). Other programs aim at reducing students' stress, anxiety, and emotional imbalances (1, 9). Studies show that children from ethnical minorities experience high levels of isolation and socio-economic disadvantages. This can have a significant impact on their mental health, leading to issues such as depression, behavioral problems, anxiety disorders, post-traumatic stress, and other difficulties (40). There is one program that focuses on learning and understanding other cultures (13). In this sense, educational systems have been playing the role of main agents for migrants' social integration without breaking with their cultures of origin (41).

We analyzed one integration program whose participants were all Arab migrants (17), aged between 20 and 30. There is another program (8) addressed to refugees coming from Africa, in which 68% of participants are aged between 21 and 40. In both interventions, participants would be included in the range of highest vulnerability for radicalization, who according to (42), are young migrants aged between 18 and 35. This leads us to assert that even though these programs do not outline radicalization prevention objectives explicitly, all programs addressed to adolescents and young migrants aiming at their integration and adaptation in the host country are preventing and driving away future radical ideas and actions from the program's participants.

### Characteristics of the Intervention

There are several programs that pair migrants with long-term residents in the city (4, 5, 6, 17). In other programs (11, 12, 18), participants talk about personal experiences and subjects of interest for the group (employment, discrimination, frustrations, and acquiring new skills). Other activities also carried out in some programs are dynamical and artistic (9, 11, 12), as well as cooking workshops (5). In one of the programs, sport activities are carried out in a mosque (16), with the aim of providing migrants with clinical and psychosocial benefits. New technologies are used in several interventions; one of the programs with students teaches about other cultures and ethnic groups through virtual activities (13). There are two programs with adult migrants that also make use of new technologies. One of them is carried out in a library (4), were there is a program called “The American Place” (TAP), designed to help migrants with public services, legal advice, classes, and employment references. The other intervention uses a smartphone application to train migrants in the pronunciation of the language (8).

### Program Assessment Method

Several programs use mixed methodologies to assess interventions (7, 8, 13, 17, 18), in conjunction with quantitative and qualitative instruments. When these instruments are used in parallel, validity, transferability, and reliability increase (43). The most widely used technique for data collection is interviews (1, 2, 3, 6, 14, 15, 16). This technique has an enormous potential to access individuals' psychology as well as their life experiences, through which their everyday nature and social relations can be discovered (44). Observation of participants is also widely used (4, 9, 11, 12). Such method enables a detailed understanding of the operation and the results of the intervention (45). (46) notes that observing participants is a process that allows researchers to learn about the study participants' activities in the natural environment through observation and by participating in their activities. One of the interventions (7) uses sociometry with students, evincing that relations between pairs play a mediating role in children and adolescents' psychosocial balance (47). This technique classifies students into four types of centrality of degree: nuclear, secondary, peripheral, and isolated (48). It is, therefore, a powerful tool to assess social skills and integration (49). It is of interest to note the importance of this tool in the context of research with migrant adolescents in schools.

Programs that carried out quantitative assessments used different questionnaires. Two of the programs carried out in schools (7, 18) used the Strength and Weaknesses Questionnaire (50), completed by both adolescents and teachers. Some programs (10, 18) also used the Self-esteem Scale (51). In another intervention (2), post-traumatic stress was measured using the Post-traumatic Diagnose Scale [PDS; (52)]. The program for the empowerment of migrant women (10) used the Behavior-Objective Scale (53) and the Treatment Satisfaction Questionnaire (54). The program for the improvement of social support (17) used the Personal Resources Questionnaire (55, 56) to measure social support perceived and satisfaction with support resources, UCLA's Reviewed Loneliness Scale (57) to assess loneliness and social isolation, and the Proactive Facing Inventory (58) to measure participants' search of emotional and instrumental support.

Most programs performed an assessment prior to the intervention and another one just after the end of it. However, some programs also used control group designs (2, 8, 14, 18). Besides pre- and post-assessments, some programs perform a follow-up after 6 months (16), after 1 month and 3 months (10), and after 1 and 2 years (14).

### Integration Programs Results

It must be noted that the results obtained in the different programs reviewed are positive, even reaching the desired objectives and with participants expressing agreement and satisfaction with interventions.

Programs addressed to migrant women show very positive results and improved women's quality of life and integration. Generally, there is a decrease in loneliness and depression (5, 10); an increase in relation networks, perception of social support, and self-esteem; and higher levels of migrants' self-confidence (11). There is also an increase in mothers' confidence in rising their children and their self-sense of ability (6). Women with post-traumatic stress who participated in the intervention showed significantly reduced symptoms, compared to the women from the control group (2), as well as experiencing a clear improvement in their symptomatology and great satisfaction with the treatment they received (10).

Programs carried out in the school and family context showed results that favor the integration of migrant children and adolescents. These programs enabled children to improve their knowledge about themselves, their culture, and that of others, as well as democratic and social justice values (13). In the intervention program for social and emotional learning with adolescents (7), students learned how to work together with their classmates and feel integrated; they felt more motivated and concentrated in all activities; they expressed their thoughts and feelings more openly, they reduced their aggressiveness, and they felt happier and with better moods. Other results from interventions with migrant adolescents show that after the intervention, they were able to reduce their stress and anxiety levels, learn coping strategies, and reduce emotional imbalances, the pain of the experience, and that of feeling different (9). The program of school therapy for migrant adolescents and refugees (18) did not report any improvements in participants' self-esteem or emotional and behavioral symptoms. However, children from the experimental group showed a significant improvement in the learning of the language and their mathematical performance compared to the control group. The program with migrant fathers in the educative context (1) also had beneficial results for participating families, as it allowed fathers to broaden their knowledge and awareness about the problems in the family and ways of helping children; communication and positive interactions between parents and children improved, as well as motivation and security feelings in children. Furthermore, trauma and skill learning in children also improved.

Programs aimed at the integration of migrants that include a relation with a long-term resident (4, 6, 17) achieved a significant reduction of participants' loneliness and increased their social support and social integration perceived, as well as an increase in the search of emotional and instrumental support. To connect recently arrived migrants with long-term residents makes their transition easier and allows recently arrived migrants to build long-term relations with migrant residents.

Language training programs (8, 14) showed positive results and enabled communication and employment search. The program aimed at improving healthcare for migrants (3) showed statistically significant results related to better quality of life. Results showed that almost 94% of participants expressed general satisfaction with the services received and access to such services; 96% expressed satisfaction with the quality and appropriateness of services received, as well as satisfaction with their participation in the program. The program aimed at improving Muslim fathers' skills was also very positively valued by participants (15).

Interventions with migrants are full of limitations; each project faced barriers or difficult situations when applied. Some programs shared the obstacle of a lack of time from the part of migrants. Some participants express having difficulties to attend programs because of their working conditions or because of being too busy with family life (1, 15). The legal status of migrants is also another aspect that must be taken into consideration, because in some occasions, for fear of deportation, some migrants hesitate to provide information or participate in such programs (2). There is another limitation caused by migrants' high levels of mobility, which makes their follow-up and long-term assessment difficult (18). Finally, some communities like Muslims, based on faith, reject participating in random studies, which greatly limits their participation (16).

## Discussion

It has been proved that there are more programs in Europe to integrate migrant population. However, longer-term programs are carried out in the US. It is possible that the US understands the need to create long-term and efficient integration programs for migrants, since it is the major receiver of migrants worldwide and the main origin of international migration flows between 2000 and 2010 (59).

It is important to bear in mind that migrants face great barriers for their integration, so the four domains noted by Suárez-Orozco and Carhill (32) must be broadened to include additional factors such as challenges of acculturation, stigmas, poverty, poor housing, psychosocial stressors, unemployment, etc. (60). It has been observed that many of these domains are not tackled in interventions with migrants so we believe they should be included in programs addressed to this collective.

There are few programs in which native population participates. It must be taken into consideration that programs exclusively addressed to migrants, rather than helping them, contribute to segregation and exclusion, as well as increasing migrants' perception of vulnerability. It would be necessary to design programs where native population and migrants participate equally and where the whole community could participate, cohabitate, and know one another.

There should be more programs in which both children and parents participate. It is key for families to have a good connection between parents and adolescents, since the family is still one of the main contexts in which social and educative values are transmitted (61). It has been proved how improving parents' coping mechanisms has a positive impact on the functioning of the family and children in low-income situations (62). Some studies show the importance of families' participation in projects, being a key element in the case of migrant children (63).

Only one program aims at integrating migrants in the field of healthcare. Some authors note that privatizing public services disconnected these services from social obligation, thus making it more difficult for individuals and institutions to have a language that can include the principles of racial justices as a common good (64). According to Dixon (65), it is necessary for a growing multicultural society to strive for honoring and respecting plural cultural world views of all individuals in the healthcare system.

School is the main contact place between migrant students and native students, which makes it the ideal place for programs that aim at promoting integration and inclusion. Students who are well-integrated in the education system of the host country, both academically and socially, are more likely to reach their potential (66). According to Haberfeld et al. (67), highly qualified migrants are more likely to migrate to labor market with broad structures of opportunities, compared to lower qualified migrants. It is therefore key for young migrants to be trained and access higher education to enter the labor market and reduce or even revert the unemployment gap (68).

In all programs analyzed, there is a general lack of use of new technologies to support migrants' integration. Pantoja and Villanueva (69) note that there are few studies that link new technologies with interculturality and even less studies with a cooperation approach. Besides that, there should also be a better diffusion of programs and interventions for migrants so the collective can know what resources are available for them.

Regarding the adaptation of programs, Falicov (70) identifies three levels of depth. The first level is translation; that is, the tools, manuals, modules, and sessions from an intervention program are translated and handed to participants in their mother tongue to increase accessibility for those migrants who are monolingual. The second level is cultural values and contextual stressors, and the third level is related to cultural theories about problem formation and therapeutic change. Most programs analyzed stay in the first level and only two of them (1, 15) reach the second level, since they are culturally adapted.

Most programs use mixed methodologies, since quantitative information does not allow for casual connections to be made (71). The most widely used technique in all programs is interviews, since it is ideal to obtain dense information. Furthermore, according to López and Deslauriers (44), interviews have a relevant complementary potential when combined with quantitative studies, since they contribute to understand participants' beliefs and experiences.

With the present work, a lack of studies that carry out programs aimed at working with the migrant collective has been observed, and only some of them perform comprehensive assessments to know whether interventions are successful and reach the desired objectives (Table 3). Against this backdrop, we understand little progress will be made to improve programs aimed at the integration of this collective.

**Table 3:** Main papers about social integration programs with migrants.

**No**.	**References**	**Location**	**Duration/funding**	**Participants**	**Objective/s**	**Characteristics of the intervention**	**Program assessment method**	**Results**
1	DeCarlo et al. (36)	US (Unspecified)	6 months. Unsubsidized.	21 participants, Latin parents. Average age: 38.07. 21 students also participated with an average age of 11.59.	To improve commitment and participation in behavioral cognitive intervention for trauma in schools, promote the development of parents' skills, and reduce migrant children's trauma.	Ten group sessions (1 h) and from one to three individual sessions (to narrate trauma) for students. One to two educational group meetings for parents (1 h). Four additional sessions for parents, including psychoeducation and communication between parents and children. These sessions had a duration of 1.5 to 2 h.	Semi-structured telephone interviews after behavioral cognitive intervention for trauma in schools with parents.	Benefits for children included the improvement of symptoms and development of skills. Parents agreed that there was a need for programs such as behavioral cognitive intervention for trauma in schools and expressed believing in the importance of parents' participation in their children's lives and school. They reported high levels of satisfaction and expressed feeling the program was beneficial for them, culturally relevant, and that they would recommend it.
2	Galano et al. (72)	US (Michigan)	10 weeks. Unsubsidized.	93 Latin women participants. Average age: 35.66.	To empower migrant women who experience gender violence to access resources and increase the efficacy of upbringing.	Group treatment of 10 sessions for adult women who experience gender violence in their community.	Women from the experimental group (53) and the control group (40) were interviewed at the start and the end of the intervention.	Women who participated in the intervention experienced a higher decrease of their post-traumatic stress disorder symptoms compared to the women from the control group. Specific reductions based on domains of symptoms were also analyzed (*p* < 0.5).
3	Paris et al. (60)	US (Connecticut)	2 years. Subsidized.	950 monolingual Latin adults, older than 18 years.	To ensure that behavioral healthcare is accessible and linguistically adapted for Latin migrants.	There were 15 focal groups with sessions of 90 to 120 min with consumers, direct services, and administrative personnel, to better understand local needs and relevant problems in order to provide quality behavioral healthcare to the Latin community.	Satisfaction survey. Five-point Likert scale. Questions are classified in five fields: general satisfaction, access, quality and appropriateness, results, and recovery.	Results showed positive experiences of consumers. 94% (*n* = 90) of interviewed reported general satisfaction with services received; 94% (*n* = 89) reported satisfaction with access to services; 96% (*n* = 90) expressed satisfaction with the quality and appropriateness of care received. Almost 96% (*n* = 90) of interviewed reported satisfaction with their participation.
4	Thomas et al. (73)	US (Connecticut)	3 years. Subsidized.	63 migrants from more than 12 countries in 4 continents. Age is not specified.	To promote the transition of recently arrived migrants.	The program paired 48 Cultural Navigators with participants. Participants used a wide range of services through The American Place (TAP), a program designed to help them access services, legal advice, and classes, as well as access to books, photographs and computers. The American Place (TAP).	Participant-observer methodology. In-depth interviews and focal groups were also carried out with the administrators of the program, personnel, volunteers, and migrant participants.	This program promoted the transition of recently arrived migrants and helped them establish relations with long-term residents. For the pairs of participants, it was relevant to talk and know each other because this created trust, security, and emotional support between them.
5	Msengi et al. (74)	US. (Midwest).	1 year. Subsidized.	15 women from different countries (Africa, Latin America, and Asia). Age is not specified.	To help migrant women who are new in the community and offer long-term residents the opportunity to meet them and better understand them.	To reduce stress and depression, participants were paired with a local female volunteer who acted as conversation partner. This allowed the migrant women to practice and improve their English command and provided them with help with their individual needs.	Mainly focal group discussions, as well as observations and assessment questionnaires after each activity.	Several participants expressed that the program helped them develop better mental and emotional well-being, as well as general well-being for them, their children, and the other members of their families. Support groups also helped these women overcome language, culture, poverty and discrimination barriers that made it very difficult for them to function in the new community.
6	Paris et al. (75)	US. (Massachusetts)	3 years maximum. Subsidized.	14 Latin female migrants. Aged between 25 and 38.	To provide support, education, and access to resources, to reduce stress levels and depression and reduce primary risk factors for child abuse.	Multilingual and bi-cultural paraprofessionals, who are migrant mothers and who convey home visits to recently arrived mothers and children. Services include weekly visits and frequent telephone contact to provide emotional and instrumental support, security, education, and resource referral.	Qualitative study. Each interview was carried out in person and had an approximate duration of 1 h. Interviewed mothers were asked to describe their experiences of arrival in the US, conditions once in the country, and their perceptions of the intervention's home visits.	Empowerment and self-esteem increased based on the relations with visitors and made mothers feel more secure in the upbringing of their children. These services also helped them feel less lonely and more connected. Home visits helped women access services that were beyond the intervention's reach.
7	Doikou et al. (76)	Europe. Greece (Thessaloniki)	2 months. Unsubsidized.	139 students (81 primary school students and 58 secondary school students). Target students were 7: 4 from Russia, 2 from repatriated Greek families, and 1 from Albania. Age range: 6–16.	To improve social behavior and integration of the target students with their classmates and provide them the opportunity to interact and know each other.	The program was carried out with the whole classroom. Twenty-nine activities were performed. During the activities, students worked in small groups from three to five children to carry out specific tasks. After each task, there was a discussion and children were asked to talk about what they thought and experienced while working together.	Qualitative and quantitative methods. Semi-structured interviews and socio-metric measures.	Results show positive interaction between target students and their classmates during activities, as well as some improvement in the behavior and social position of target students after the completion of the intervention.
8	Bradley et al. (77)	Europe (Sweden)	10 weeks. Subsidized.	38 migrants (12 women and 26 men). Most participants were Syrian (35). Age range: 20–60 years, most participants were aged between 20 and 30.	To explore how access to mobile technology can be used to integrate recently arrived migrants, through language and culture training.	The control group (14 participants) received traditional training and the experimental group (24 participants) used a mobile application to learn pronunciation as a complementary activity to the program. It combined teaching and self-study. Three hours, twice a week. The program includes subjects related to the Swedish labor market, the economy, civic orientation, culture, and language.	Mixed methods. Participants were interviewed about their command in the use of smartphones and they were given some activities to perform in and out of the classroom to compare their linguistic evolution. Interviews, weekly activity reports, and observations were also carried out. Participants also did weekly online self-assessments for their mobile activities outside the classroom.	Participants used smartphones mainly to communicate with their families and friends instead of with Swedish people and learn Swedish. Compared to the control group, the experimental group showed faster language learning and higher self-confidence to speak the language. The study concludes that language training with a pronunciation mobile application is very useful to develop spoken language skills, which can help reach better integration for migrants.
9	Rico et al. (78)	Europe. Spain (Madrid)	6 years. Subsidized.	Migrant children and adolescents. Number of participants and origin are not specified. Age range: 6–18.	To prevent exclusion and promote integration.	Flexible sessions, including photography, painting, poetry and cinema workshops and debates.	Assessment reports of creativity workshops, participants' assessment files, and communication files with the educational space.	Assessment reports provide positive data on the functioning of the workshop in the following fields: reducing stress and anxiety, reducing emotional imbalances due to participants' circumstances, interest for people outside their cultural groups and reducing the pain of the migrating experience and the feeling of being different.
10	Salaberria et al. (79)	Europe. Spain (Basque Country)	5 months. Unsubsidized.	5 Latin American women (2 from Honduras, 1 from Bolivia, 1 from Colombia, and 1 from Venezuela). Average age: 28.6 (range: 23–35).	To empower migrant women and provide them with skills to face psychological distress, as well as help them achieve their goals in life.	Psychological support program. It includes 8 weekly individual sessions of 1.5 h, with cognitive-behavioral support.	Pre-treatment assessment (two to three sessions). The psychological support program was subsequently applied, and once finished, post-treatment assessment and follow-ups were carried out after 1 and then 3 months.	Women experienced a clear improvement of psychopathological symptoms in general. This improvement is clear after comparing pre-treatment and post-treatment assessments, as well as the achievements obtained, which can be seen 1 and 3 months after the intervention. The level of difficulty perceived by women to achieve their goals in life and improve their quality of life is considerably reduced when comparing pre-treatment and post-treatment assessments. Such levels of difficulty continue to reduce after 3 months. Participants also report high levels of satisfaction with the help received.
11	Carrascosa (80)	Europe. Spain (Madrid)	3 months. Unsubsidized.	6 women from Latin America and 1 from Africa. Age range: 30–49.	To create a space of self-expression where participants feel free to express, share, give and receive.	Art creation workshop. One weekly session with a duration of 2 h. Different proposals were suggested to create individual and group dynamics, as well as crafts and talks.	Direct observation assessment. The behavior of participants was observed during workshops.	It was observed that as participants felt more confident in the group, they reduced their insecurity. Security was created little by little, whether this was by the way they handled the materials, which materials they chose or in the explanation of their work. The group created a safe space for creation, experimentation, dialogue, and commitment between participants.
12	Bravo et al. (81)	Europe. Spain (Logroño)	Unspecified duration. Unsubsidized.	12 women (8 from Morocco and 4 from Algeria, Gambia, Pakistan and Russia). Age range: 23–50.	To promote migrant women's social integration, support networks, and increase self-esteem.	One weekly session where an altruistic exchange space is created between people with same concerns, similar circumstances, and needs. Dynamics, video watching, talks, etc.	Assessment based on “brainstorming” through opinion exchange groups and participants' observation, thus giving rise to a field journal.	Women expressed high enthusiasm with meetings, of which they said they would not change anything. The program helped empower women, the creation and strengthening of bonds between them, and the broadening of their points of view.
13	Pantoja et al. (69)	Europe. Spain.	9 months. Unsubsidized.	532 Spanish students. Age range: 11–12.	To promote interaction and cultural knowledge between students from different countries.	The program was designed in a virtual environment, where children from Latin America, Portugal, and the UK interact. Its psycho-pedagogical principles are development of ethnic and cultural identity of students, cultural pluralism, and education for citizenship. Reflection and personal critical analysis.	Mixed methodologies. Quantitative through the application of a Likert-type scale and qualitative trough portfolios and classroom reports.	The program provided students with valuable information about dengue fever, nature, meeting different people, and other cultures. Students also learned about monuments (Latin American students were surprised as there are not similar buildings in their environments), about the different currencies, and about languages.
14	Tsoukalas et al. (37)	Europe (Greece)	6–8 months. Subsidized.	260 migrants. Average age: 38.58.	To learn Greek language, tackle the issue of lack of communication between natives and migrants, and prevent their exclusion from the labor market, and, therefore, society.	The experimental group included 110 participants and the control group included 150 participants. Training programs are structured in four learning levels: Levels A, B, and C have a duration of 100 h and level D has a duration of 125 h. The additional 25 h cover Greek history and culture lessons. Participants learn the Greek language, which allows them to communicate with natives even from the first levels of the program.	An initial test was carried out on the same year of the training, a second one was carried out after 1 year, and a third one was carried out after 2 years from receiving training.	Results show that the positive effects of the training in migrants' integration are clear 2 years after completing the program. Migrants who participated in the Greek language courses have a higher income as compared to the equivalent sample in terms of demographical features of untrained migrants.
15	Scourfield et al. (82)	Europe (South of England)	2 months. Subsidized.	5 Muslim fathers. 4 ethnically original from Pakistan and 1 from India. Age is not specified.	To improve emotional and cognitive issues of fathers and promote their increasing use of positive skills.	The intervention is based on psychoeducational and cognitive-behavioral approaches of learning. The course comprises 10 group and interactive sessions of a duration of 2 h. Role plays and testing strategies in the home environment are used, with religious texts that support the main messages of the course.	Qualitative small-scale study, with approximately 25 h of observation of participants, focusing on the interaction of participants before and after group meetings, and 13 structured interviews. The wives of the 5 participants, who had attended the program for mothers also participated.	Each father highlighted different subjects that they considered had been the main learning point for him. This list included learning about “self-power to handle family affairs” and implement strategies to handle behaviors, cooperation between husband and wife to calm children when they are angry, to control emotions, to show empathy and connect with their children, and to spend more time and share concerns with their wives. Interviewed women talked about the changes in their husbands' attitudes toward themselves and the children, less anger, and more empathy. Some had seen positive changes in their children after fathers' participation in the program.
16	Banerjee et al. (83)	Canada (Toronto)	6 months. Unsubsidized	62 Muslim women. Average age: 51.	To allow women to develop physical activity patterns and empower them.	The intervention included training group workouts that included aerobic and strength training. Three weekly evening of a duration of 1 h in a mosque.	This intervention was a small-scale pilot project based in one community with one group and two assessments, a previous one (3 Likert-type questions) and a later one carried out 6 months after completion of the program (12 Likert-type questions).	Nineteen participants agreed to the previous and posterior assessments. Participants considered the workout classes positive, convenient, easy to follow, educative, useful, and supportive.
17	Stewart et al. (84)	Canada (Toronto and Edmonton)	3 months. Unsubsidized.	58 refugees from Africa. Age range: 18-54.	To improve social support and integration of African refugees.	Support group included 12 participants each. Each group met twice per week in one session in person. Each session lasted from 60 to 90 min and telephone sessions lasted approximately 20 min. Participants chose subjects for each session. Key subjects that were debated included improving cultural understanding and social integration, navigating the system, developing new skills, optimal employment search, improving family dynamics, and overcoming racism and discrimination.	Qualitative and quantitative methods. Participants completed the same quantitative measures before the intervention (initial test) and after (final test). Group interviews were also conducted using an interview guide.	There were significant increases in support received (*p* = 0.002), social integration (*p* = 0.002), and significant reductions in loneliness (*p* = 0.002). After the intervention, participants reported that they had learned how to look for services and support and how to face challenges.
18	Rousseau et al. (85)	Canada (Montreal)	9 weeks. Unsubsidized.	123 adolescents from Asia, Eastern Europe, Latin America, Middle West, and Africa. Age range: 12–18.	To provide young migrants and refugees the opportunity to empower and share group stories, support the construction of meaning and identity, and create a bridge between the past and the present.	Nine weekly sessions with a duration of 75 min. Each week, a different topic is presented based on which each participant tells a brief personal experience. They are subsequently invited to express their experiences or concerns about the topic using fluid sculptures, diatribes, pairs, and other reflexive techniques.	Qualitative and quantitative methods. Data were collected before and after the intervention. Assessment questionnaires were conducted before and after the program.	Adolescents' general perception of disability decreases significantly as well as the interference of these symptoms in friendships, family life, and leisure activities. School performance also improved, particularly in mathematics and French speaking in both the experimental and control group.

It has been observed that there is a lack of resources, or that the existing ones are very limited, when it comes to carrying out the programs; only seven of them were subsidized by the State where they were being carried out. This evinces that the economic support given to these programs is still insufficient, even though integration is an issue that deeply affects intercultural cohabitation. It would be necessary, therefore, to improve social policies to reduce and/or avoid the social consequences brought about by migrating processes that have political, demographic, socio-economic, and cultural impacts.

It must be highlighted that there is a lack of intervention with Muslim migrants of second and third generation, even though second generations of the Muslim diaspora have been particularly vulnerable to processes of jihadist radicalization (86). Interventions to promote the integration of these young migrants is necessary. It would also be convenient to use culturally relevant structured networks of these young migrants, such as mosques, since they are important assets when designing interventions. Likewise, it would be of great importance to carry out community interventions due to the polarization that occurs regarding religion and cultures, with the objective of avoiding ethno-violence and racism, for instance, when there is news of a Muslim attack in some country.

It has been observed that some of the most effective activities are those that group recently arrived migrants with native population and/or long-term residents. The latter act as mentors and translators, resource guides, and teachers, helping recently arrived migrants understand and know the resources available, feel more secure and less isolated in the new country, and thus helping to reduce prejudices and discrimination from the part of native population. Furthermore, results show the importance of language training for migrants, which should be a priority for host countries. To work on intercultural education is also important, since not only is it a “complement” to the standard education plan, but also, according to the United Nations Education, Science and Culture Organization (87), the development of inclusive study plans that include language, history, and culture training of non-dominant groups of society is necessary.

It is important to highlight the positive results of those programs focused on migrant women. Today, there is talk of a “feminization of migration,” even though female migration is not a new phenomenon (88). We consider it necessary to integrate the gender approach in all migratory policies with the aim of promoting the integration and adaptation of host countries to this vulnerable collective.

Results from the present research allow us to identify key elements when it comes to carrying out programs for the integration of migrants. Our results suggest that future studies of programs should be more detailed and specify data such as origin, number of participants, age of participants, and activities carried out. Besides, programs should also perform more exhaustive assessments as well as develop in a more extensive and detailed manner citizen participation and community work in social cohesion interventions. Programs aimed at the integration of migrants contribute to decrease feelings of lack of roots and identity and, subsequently, as pointed out by Heelsum and Vermeulen (17), the search of an alternative group with more radical ideas. Therefore, it is necessary to develop integration programs that would satisfy the needs of migrants. This could be done by using new technologies and community facilities, along with participation in the community. We would like to note the importance of working with students at schools, given the fact that most vulnerable migrants to radicalization are those from younger generations who are born in host countries. It is, therefore, essential to work with children and adolescents in order to avoid their exclusion from the system, which could lead to potential radicalization.

There were several limitations during the present research. One of them was the lack of programs that focus on the integration of migrants, despite being an issue of concern for countries. It must be noted that some studies analyzed do not specify the origin of migrants or their age, and most interventions do not detail activities carried out. Another limitation of the study is that some programs do not have systematic assessments in place and, sometimes, do not mention who carries out the said assessment. If the team responsible for carrying out the intervention is the same as the one carrying out the assessment, results can become contaminated. This means that the conclusions of the present study are limited when it comes to how the programs analyzed work, due to the limited information available from such programs.

Through this study, we have confirmed the great importance of integration programs in countries with increasing flows of migration, since the benefits include an improvement in migrants' quality of life. Likewise, these programs also benefit host countries, since integrating migrants prevents extreme and radical actions and favors intercultural cohabitation.

## Data Availability Statement

The original contributions presented in the study are included in the article/supplementary material, further inquiries can be directed to the corresponding author/s.

## Author Contributions

NDP-B, IH-M, and LG-J contributed to conception and design of the work, substantial contributions to revising the work critically, and wrote the manuscript. AG-C and MM-F wrote the manuscript. All authors involved approved the final version of the manuscript to be published.

## Conflict of Interest

The authors declare that the research was conducted in the absence of any commercial or financial relationships that could be construed as a potential conflict of interest.

## Publisher's Note

All claims expressed in this article are solely those of the authors and do not necessarily represent those of their affiliated organizations, or those of the publisher, the editors and the reviewers. Any product that may be evaluated in this article, or claim that may be made by its manufacturer, is not guaranteed or endorsed by the publisher.
